# circRNA-programmed macrophages enable localized antibody therapy

**DOI:** 10.1016/j.omtn.2026.103010

**Published:** 2026-07-21

**Authors:** Yawei Du, Daoyu Zhu, Shifeng Ling, Yixuan Chen

**Affiliations:** 1 Department of Orthopaedics, Shanghai Key Laboratory for Prevention and Treatment of Bone and Joint Diseases, Shanghai Institute of Traumatology and Orthopaedics, Ruijin Hospital, Shanghai Jiao Tong University School of Medicine, 197 Ruijin 2nd Road, Shanghai 200025, P.R. China; 2 Department of Orthopaedics, Shanghai Sixth People’s Hospital Affiliated to Shanghai Jiao Tong University School of Medicine, 600 Yishan Road, Shanghai 200233, P.R. China

## Main Text

Unlike traditional infectious-disease vaccines, mRNA therapeutics offer a far broader application landscape. Among various next-generation mRNA strategies, protein-replacement therapy is particularly promising.^1^ Although antibodies are widely used clinically, systemic administration often entails notable systemic adverse effects and treatment burden. By contrast, using mRNA to encode and synthesize antibodies *in situ* within the body confers multiple advantages: (1) achieving sufficient *in vivo* expression at lower exogenous doses, thereby increasing effective exposure and bioavailability; (2) a highly modular, rapidly iterable manufacturing process with clear cost and scalability benefits; (3) the ability, via delivery systems, to target specific cells or tissues, improving tissue penetration and local action while reducing off-target effects.^2^^,^^3^ Unlike linear mRNA, circular RNA (circRNA) possesses a covalently closed loop lacking 5′ and 3′ ends, which effectively protects it from exonuclease degradation, resulting in greater stability and lower innate immunogenicity.^4^^,^^5^ Moreover, it can sustain protein production through internal ribosome entry site (IRES)- or m6A-mediated translation. Consequently, circRNA offers distinctive and important advantages for *in situ* antibody synthesis and protein-replacement therapy.

**Figure 1:**
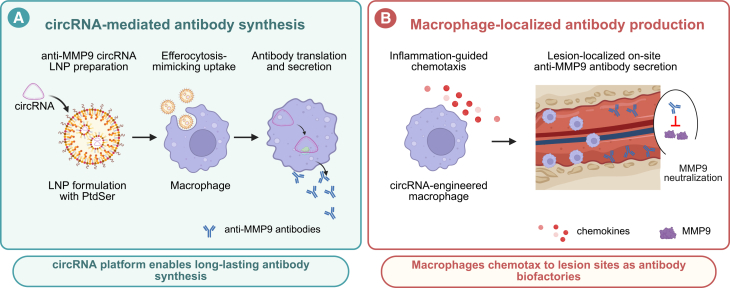
Schematic of circRNA-programmed macrophages enable localized antibody therapy (A) Antibodies are encoded by circRNA and packaged in optimized LNPs. Upon incorporation of PtdSer, the LNPs are taken up by monocytes/macrophages via an enhanced efferocytosis-mimicking effect, enabling long-lasting *in vivo* expression. (B) circRNA-engineered macrophages exhibit innate chemotaxis toward inflammatory lesions and are particularly active in osteoclast-related processes, resulting in a distinctive *in vivo* antibody distribution and high accumulation at disease sites (created with BioRender.com, with permission).

In our recently published study in *Bioactive Materials*, clinical data from patient blood and bone tissues revealed pathological upregulation of matrix metalloproteinase-9 (MMP9), suggesting that antibody therapies targeting MMP9 may ameliorate degenerative bone disease phenotypes (Figure 1).^6^ Given the active involvement of macrophages within degenerative bone lesions, we proposed a macrophage-targeted circRNA delivery strategy to drive on-site expression of anti-MMP9 antibodies as an efficient on-site antibody-replacement approach. To enhance specific recognition and uptake by monocytes/macrophages, we incorporated phosphatidylserine (PtdSer) into the lipid nanoparticle (LNP) formulation to mimic apoptotic cues.^7^^,^^8^ Simultaneously, we moderately reduced the polyethylene glycol (PEG)-lipid fraction to further increase affinity for and endocytosis by monocytes/macrophages. These formulation optimizations markedly improved monocyte/macrophage uptake and supported more durable *in vivo* expression of the anti-MMP9 antibody. *In vivo*, the innate inflammation-guided chemotaxis of macrophages facilitates the systemic, targeted distribution of this on-site antibody synthesis modality, thereby further amplifying therapeutic efficacy.

In an aged mouse model, macrophage-targeted delivery of anti-MMP9 circRNA therapy validated the translational potential of this approach: the tissue-level senescence-associated secretory phenotype (SASP) was markedly suppressed, accompanied by downregulation of P21 and MMP3, indicating a rebalanced interplay between osteoblasts and osteoclasts. These *in vivo* findings indirectly support the pathological significance of the MMP9 overexpression observed in clinical specimens and underscore MMP9 as a potential therapeutic target for degenerative bone diseases. As a key effector within the tissue microenvironment, precise modulation of MMP9 is critical for attenuating the inflammation-senescence axis and restoring bone remodeling homeostasis. By contrast, conventional systemically administered antibodies often lack tissue and cell-type specificity, exhibit limited tissue penetration, and lead to high systemic exposure, thereby increasing off-target effects and adverse events. Leveraging macrophages as cellular vehicles for *in situ* circRNA expression enables targeted and sustained neutralization of MMP9 within inflamed microenvironments, reducing systemic exposure and widening the therapeutic window, thus offering a more precise intervention for degenerative bone disease. Undoubtedly, these advantages of circRNA-based therapy are not achievable with traditional antibody drugs.

The applications of circRNA-based antibody replacement therapy are not limited to intravenous (i.v.) macrophage engineering, nor solely to the treatment of degenerative bone diseases. In another study, we integrated circRNA with an artificial vascular stent to achieve rapid, selective transfection of local endothelial cells after implantation, thereby inducing secretion of Olaratumab and modulating the phenotype of adjacent smooth muscle cells, which reduced the risk of restenosis.^9^ This case illustrates that circRNA-driven *in situ* antibody expression possesses high programmability and contextual adaptability: its design space and application scope extend well beyond the administration and distribution paradigms of traditional antibody drugs, offering a scalable pathway for precision biotherapeutics across multiple tissues and disease settings.

There are still some limitations of this circRNA framework via encoding conventional antibodies. Specifically, the expression ratio of heavy and light chains, as well as transcript architecture and ordering, profoundly affects *in vivo* translation, secretion, and assembly, thereby constraining antibody yield and functional consistency. Looking ahead, we will focus on smaller, structurally simpler formats such as single-chain variable fragments (scFvs) and nanobodies (single-domain antibodies).^10^ scFvs, which tether variable heavy and light domains via a flexible linker, provide high expression plasticity and facilitate construction of bivalent or bispecific formats. However, their *in vivo* stability and aggregation propensity require further optimization through linker and framework engineering. Nanobodies, owing to their single-domain architecture, high thermal stability, capacity for high-density expression in immune cells, and favorable tissue penetration, represent priority candidates for the circRNA platform.

Overall, we propose a novel circRNA-based antibody replacement therapy. By targeting macrophages and leveraging their innate chemotaxis toward inflammatory and degenerative lesions, we optimize the *in situ* (on-site) synthesis of antibodies for improved *in vivo* distribution and tissue enrichment. This approach has been preliminarily validated in a degenerative bone disease model targeting MMP9, demonstrating promising feasibility and translational potential.

## Acknowledgments

This work was supported by the 10.13039/501100001809National Natural Science Foundation of China (82572422 and 32500658), Ruijin Hospital mRNA Drug Basic and Translational Research Project (JZ202413), and the 10.13039/501100008233Shanghai Jiao Tong University School of Medicine “Clinical Full-time Research Team” Project (20250402).

## Declaration of interests

The authors declare no competing interests.

## Declaration of generative AI and AI-assisted technologies in the writing process

During the preparation of this manuscript, the authors used ChatGPT (GPT-5, OpenAI) to assist with language refinement, including grammar correction and improvement of clarity and readability. The tool was not used to generate scientific ideas, analyze data, or draw conclusions. All text produced with AI assistance was critically reviewed and revised by the authors to ensure scientific accuracy and integrity. The authors take full responsibility for the final content of the manuscript.
